# Dissection dimensions differ: Global variability in ascending aortic size challenges universal surgical thresholds

**DOI:** 10.1016/j.xjse.2026.100124

**Published:** 2026-05-04

**Authors:** Nimrat Grewal, Nora Bacour, Mohammad Zafar, Simran Grewal, Mohammed Idhrees, Bashi Velayudhan, Julia Dumfarth, Simone Gasser, John Elefteriades

**Affiliations:** a Department of Cardiothoracic Surgery, Amsterdam University Medical Center, Amsterdam, The Netherlands; b Department of Cardiothoracic Surgery, Aortic Institute at Yale New Haven Hospital, Yale University School of Medicine, New Haven, Conn; c Department of Orthopaedic Surgery, Onze Lieve Vrouwe Gasthuis, Amsterdam, The Netherlands; d Department of Cardiothoracic Surgery, Institute of Cardiac and Aortic Disorders, SRM Institutes of Medical Science, Chennai, India; e Department of Cardiac Surgery, Medical University of Innsbruck, Innsbruck, Austria

**Keywords:** aortic dissection, ascending aorta, risk stratification, sex differences, geographic variation, surgical thresholds

## Abstract

**Background:**

Current surgical thresholds for ascending aortic replacement are derived from Western, male-predominant populations. Whether these thresholds apply across diverse ethnic and geographic groups remains uncertain. We evaluated global and sex-specific variability in ascending aortic dimensions at the time of acute type A aortic dissection (ATAAD).

**Methods:**

We retrospectively analyzed 1388 surgically treated ATAAD patients from 4 international cohorts: Netherlands (n = 500), United States (n = 382), Austria (n = 435), and India (n = 71). Ascending aortic diameters were measured on preoperative contrast-enhanced computed tomography scans. Multivariable linear regression adjusted for age, sex, body surface area, hypertension, and diabetes.

**Results:**

Age at dissection differed markedly by region, with Indian patients presenting more than a decade earlier than Western cohorts (48.5 years vs ∼63 years). Women presented later than men (68 years vs 59 years; *P* < .001) but had smaller ascending aortic diameters (mean, 46.0 ± 9.8 mm vs 48.4 ± 10.6 mm; *P* < .001). Country of origin independently predicted aortic size; compared with Dutch patients, Indian (B = +7.09 mm; *P* < .001), American (B = +3.11 mm; *P* < .001), and Austrian (B = +2.42 mm; *P* < .001) cohorts had larger diameters. Only 19.5% dissected at diameters ≥55 mm, while 39.7% presented between 45 and 54 mm. Because acute dissection may transiently enlarge the aortic lumen, measured diameters likely overestimate predissection size.

**Conclusions:**

Ascending aortic dimensions at ATAAD vary substantially by sex and geographic origin. Most dissections occur below current surgical thresholds, indicating that absolute diameter alone inadequately defines risk. These findings challenge the universality of fixed cut-offs and support the need for population- and sex-specific approaches to aortic risk assessment.

**Figure:**
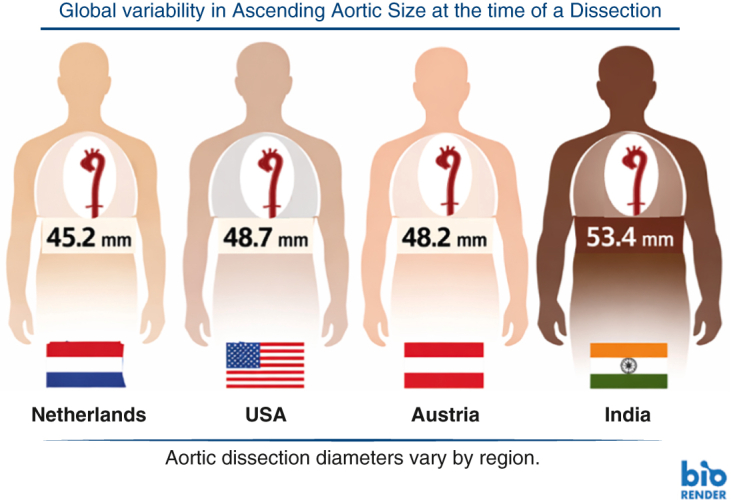
Geographic variation in ascending aortic size at the time of ATAAD.

**Central Message**

Ascending aortic size at type A dissection varies by sex and geographic origin, with most events occurring below current surgical thresholds, challenging the universality of fixed diameter cut-offs.

**Perspective**

In a cohort of 1388 patients with acute type A aortic dissection drawn from across Europe, North America, and South Asia, ascending aortic size at dissection differed by sex and geography. Most dissections occurred below current prophylactic thresholds. These findings call into question the use of a single absolute diameter cut-off and support consideration of sex- and population-specific approaches to aortic risk assessment.

Acute type A aortic dissection (ATAAD) remains one of the most catastrophic cardiovascular emergencies, with mortality rising by the hour if untreated.^1^^,^^2^ Prophylactic replacement of the ascending aorta is central to prevention.^3^^,^^4^ Current American and European guidelines recommend elective surgery once the ascending aortic diameter reaches 50 to 55 mm in patients without genetic syndromes, or 45 mm in selected high-risk groups.^3^^,^^4^ Yet landmark data from the International Registry of Acute Aortic Dissection revealed that most patients dissect below these thresholds, the so-called “aortic size paradox.”^5^ While this observation reshaped our understanding of risk, an underlying assumption that a single set of diameter-based cut-off values applies equally across all populations has persisted.

This assumption is being increasingly challenged, however. Reference values for aortic size stem largely from Western, male-predominant cohorts and might not capture the normal physiologic variability that exists across sex and ancestry.^6^ The literature, although scant, has revealed substantial interethnic and intersex variations in aortic size in the general population.^7^, ^8^, ^9^ In particular, prior work has shown that women and individuals of Asian descent typically have smaller baseline aortas, even after adjusting for body size.^6^^,^^8^^,^^10^, ^11^, ^12^

Consequently, a diameter of 45 mm may represent a far greater degree of dilation, and possibly higher relative wall stress, in these groups compared to that in large-framed Western men. Evidence comparing actual dissection diameters across continents has been virtually absent, however.

India, now the world's most populous nation, exemplifies this knowledge gap; data on thoracic aortic dimensions and dissection characteristics remain limited to small, single-center studies.^9^^,^^13^^,^^14^ Without global comparative analyses, it remains unclear whether the “aortic size paradox” manifests uniformly or whether population-specific factors modulate the diameter at which catastrophic rupture occurs.

To address this question, we conducted the first cross-continental analysis of ascending aortic dimensions at the time of ATAAD, encompassing cohorts from Europe, North America, and South Asia. By harmonizing imaging protocols across 1388 surgically treated patients, we sought to characterize geographic variability in aortic size at dissection and to evaluate whether current surgical thresholds adequately reflect global risk.

## Methods

### Data and Ethical Considerations

This study was conducted at using data from 4 countries: India, Austria, The Netherlands, and the United States. Data from India were collected from the SRM Institutes for Medical Science (SIMS Hospital) in Chennai; data from Austria were collected from the Medical University of Innsbruck; data from The Netherlands were collected from the Amsterdam University Medical Center; and data from the United States was collected from Yale New Haven Hospital. The local Medical Ethical Committee of each hospital reviewed the study proposal and waived the requirement for patient informed consent (reference METC 2024.0120; UN5106; approved April 24, 2024).

### Study Population

**Figure 1:**
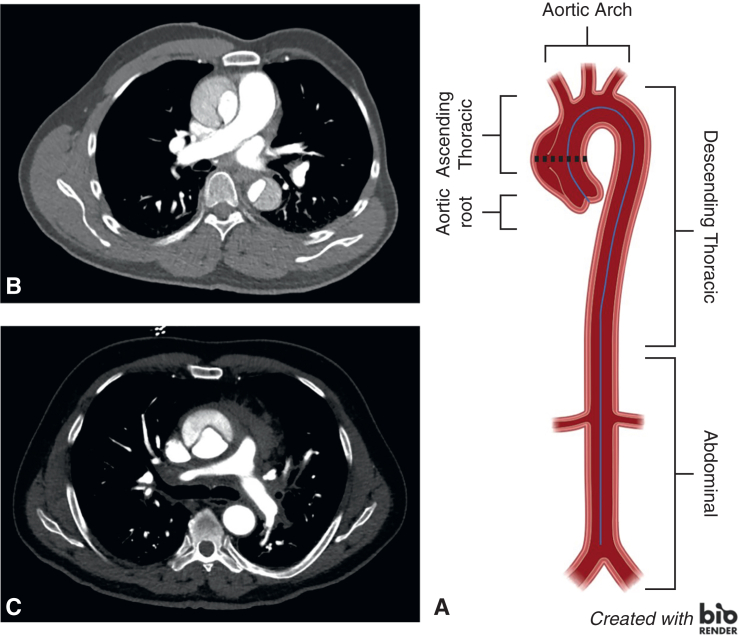
A, Schematic representation of the thoracic aorta illustrating the segment of interest and the location of maximal ascending aortic diameter measurement. B-C, Representative axial contrast-enhanced computed tomography images demonstrating measurement of the ascending aorta using centerline-based multiplanar reconstruction and an orthogonal imaging plane.

To assess whether current geometric guidelines are equally valid and applicable across different populations, we included patients diagnosed with ATAAD between 2000 and 2025. Ascending aortic diameters were measured on contrast-enhanced computed tomography (CT) angiography using centerline-based multiplanar reconstruction. The entire ascending aorta, from the sinotubular junction to the origin of the innominate artery, was systematically evaluated, and the maximal ascending aortic diameter within this segment was recorded by 2 researchers from each of the 4 participating institutions^15^ (Figure 1). Measurements were performed in a plane orthogonal to the vessel centerline to ensure accurate cross-sectional assessment. Most patients presented with acute dissection as their first diagnostic imaging study. Predissection imaging was not systematically available; therefore, measurements were done uniformly on the CT scan obtained at the time of diagnosis.

**Figure 2:**
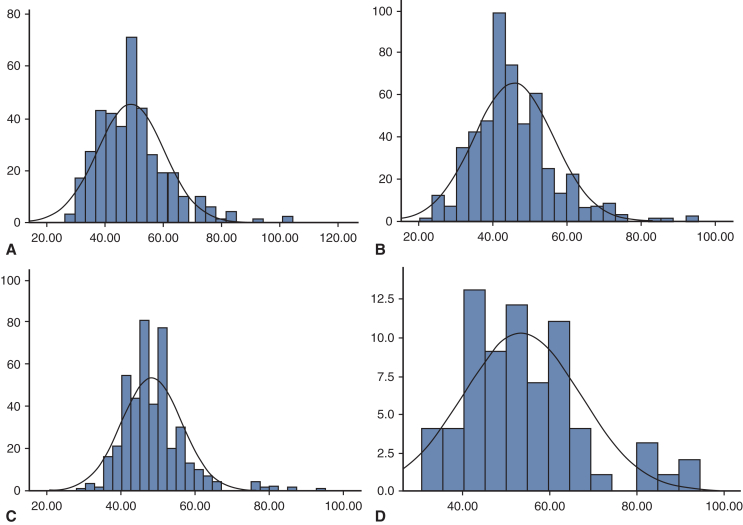
Ascending aortic diameter distribution shown in centers from the United States (A), the Netherlands (B), Austria (C), and India (D).

Patients with isolated intramural hematoma without an identifiable intimal tear and retrograde type A dissection originating from the descending thoracic aorta were excluded from the analysis.

Patient data were obtained from digital health records and hospital databases, including baseline characteristics such as age, sex, height, body mass index, and cardiovascular-related comorbidities. Patients age >18 years were included in the study cohort.

### Statistical Analysis

Before commencing data analysis, all variables were examined to assess their distribution and normality. Normally distributed continuous variables are presented as mean ± standard deviation, and non-normally distributed continuous variables are presented as median with interquartile range. Categorical variables are presented as frequency (n) and percentage.

Comparisons between 2 study groups were performed using an independent *t* test for normally distributed continuous data and the Mann-Whitney *U* test for non-normally distributed continuous data. Comparisons between multiple study groups were performed using one-way analysis of variance for normally distributed continuous data and the Kruskal-Wallis test for non-normally distributed continuous data. Categorical data were analyzed using the χ^2^ test or Fisher exact test, depending on whether the assumption of sufficient cell counts was met.

Finally, multivariable linear regression analysis was conducted to examine variability in aortic dimensions at dissection, adjusting for sex, age, height, and clinically relevant comorbidities (ie, hypertension and diabetes mellitus).^16^ A 2-sided *P* value <.05 was considered statistically significant. All analyses were performed using SPSS version 30.0 (IBM).

## Results

### Patient Characteristics

This study investigated 1388 cases of ATAAD across 4 medical centers worldwide: the Netherlands, n = 500 (36.0%), 39.8% female, median age 63.0 years; United States, n = 382 (27.5%), 37.4% female, median age 63.0 years; Austria, n = 435 (31.3%), 33.8% female, median age 61.0 years; and India, n = 71 (5.1%), 12.7% female, median age 46.0 years (Table 1). Regarding sex-related differences, the mean age at presentation varied significantly, with females presenting nearly a decade later than males in all countries (68.0 years vs 59.0 years; *P* < .001) (Table 2).

**Table 1:** Baseline patient characteristics

Characteristic	Total (N = 1388)		United States (N = 382)		The Netherlands (N = 500)		Austria (N = 435)		India (N = 71)		*P* value
Age, y, median (IQR)	62.0 (51.0-72.0)		63.0 (53.0-73.3)		63.0 (53.0-72.0)		61.0 (51.0-71.0)		46.0 (36.0-59.0)		<.001
Female sex, n (%)	498 (35.9)		116 (37.4)		199 (39.8)		147 (33.8)		9 (12.7%)		<.001
Height, cm, mean ± SD	173.9 ± 10.5	144∗	172.6 ± 11.4	26∗	175.4 ± 10.6	61∗	174.3 ± 9.3	17∗	168.4 ± 10.2	10∗	<.001
Weight, kg, mean ± SD	83.0 ± 20.7	97∗	88.1 ± 27.2	22∗	80.9 ± 16.1	51∗	81.8 ± 18.1	14∗	76.4 ± 16.9	10∗	<.001
BMI, kg/m^2^, mean ± SD	27.4 ± 6.1	144∗	29.6 ± 8.3	26∗	26.3 ± 4.7	61∗	26.9 ± 4.8	17∗	26.9 ± 5.4	10∗	<.001
BSA, m^2^, mean ± SD	2.0 ± 0.3	114∗	2.0 ± 0.3	26∗	2.0 ± 0.2	61∗	2.0 ± 0.2	17∗	1.9 ± 0.2	10∗	<.001
AHI, mean ± SD	2.7 ± 0.6	114∗	2.8 ± 0.7	26∗	2.6 ± 0.6	61∗	2.8 ± 0.5	17∗	3.2 ± 0.9	10∗	<.001
Connective tissue disease, n/N (%)	57/1385 (4.1)		20/382 (5.2)		26/500 (5.2)		9/435 (2.1)		2/68 (2.9)		.055
Hypertension, n/N (%)	742/1315 (56.4)		219/334 (65.6)		178/500 (35.6)		299/416 (71.9)		46/65 (70.8)		<.001
Diabetes mellitus, n/N (%)	92/1324 (6.9)		34/334 (10.2)		10/500 (2.0)		34/425 (8.0)		14/65 (21.5)		<.001
Dyslipidemia, n/N (%)	216/1177 (18.4)		131/333 (39.3)		21/500 (4.2)		63/282 (22.3)		1/62 (1.6)		<.001
Current tobacco use, n/N (%)	298/891 (33.4)		146/241 (60.6)		72/385 (18.7)		66.204 (32.4)		14/61 (23.0)		<.001
COPD, n/N (%)	99/1312 (7.5)		44/332 (13.3)		25/500 (5.0)		26/418 (6.2)		4/62 (6.5)		<.001
Known AAA, n/N (%)	119/1227 (9.7)		57/332 (17.2)		56/454 (12.3)		3/434 (0.7)		3/7 (42.9)		<.001
BAV, n/N (%)	67/1345 (5.0)		15/382 (3.9)		22/500 (4.4)		25/406 (6.2)		5/57 (8.8)		.242

*IQR*, Interquartile range; *SD*, standard deviation; *BMI*, body mass index; *BSA*, body surface area; *AHI*, aortic height index; *COPD*, chronic obstructive pulmonary disease; *AAA*, abdominal aortic aneurysm; *BAV*, bicuspid aortic valve.

∗ Number of missing values.

**Table 2:** Baseline characteristics by sex

Characteristic	Total (N = 1388)		Male (N = 890)		Female (N = 498)		*P* value
Age, y, median (IQR)	62.0 (51.0-72.0)		59.0 (49.0-69.0)		68.0 (57.3-75.0)		<.001
Height, cm, mean ± SD	173.9 ± 10.5	114∗	177.4 ± 8.8	70∗	166.9 ± 15.3	44∗	<.001
Weight, kg, mean ± SD	83.0 ± 20.7	97∗	87.9 ± 20.8	58∗	74.1 ± 17.2	39∗	<.001
BMI, kg/m^2^, mean ± SD	27.4 ± 6.1	114∗	28.0 ± 6.3	70∗	26.4 ± 5.7	44∗	<.001
BSA, m^2^, mean ± SD	2.0 ± 0.3	114∗	2.0 ± 0.2	70∗	1.8 ± 0.3	44∗	<.001
AHI, mean ± SD	2.7 ± 0.6	114∗	2.7 ± 0.6	70∗	2.8 ± 0.3	44∗	.553
Connective tissue disease, n/N (%)	57/1385 (4.1)		35/887 (3.9)		22/498 (4.4)		.674
Hypertension, n/N (%)	742/1315 (56.4)		495/842 (58.8)		247/273 (52.2)		.024
Diabetes mellitus, n/N (%)	92/1324 (6.9)		60/848 (7.1)		32/476 (6.7)		.823
Dyslipidemia, n/N (%)	216/1177 (18.4)		142/746 (19.0)		74/431 (17.2)		.436
Current tobacco use, n/N (%)	298/891 (33.4)		196/564 (34.8)		102/327 (31.2)		.303
COPD, n/N (%)	99/1312 (7.5)		60/837 (7.2)		39/475 (8.2)		.515
Known AAA, n/N (%)	119/1227 (9.7)		79/775 (10.2)		40/452 (8.8)		.485
BAV, n/N (%)	67/1345 (5.0)		54/859 (6.3)		13/486 (2.7)		.004

Percentages are calculated based on the number of patients with available data for each variable. *IQR*, Interquartile range; *SD*, standard deviation; *BMI*, body mass index; *BSA*, body surface area; *AHI*, aortic height index; *COPD*, chronic obstructive pulmonary disease; *AAA*, abdominal aortic aneurysm; *BAV*, bicuspid aortic valve.

∗ Number of missing values.

In terms of comorbidities, hypertension was highly prevalent across the cohort (56.4%), particularly among patients from the United States (65.6%), Austria (71.9%), and India (70.8%), while the prevalence was notably lower in the Netherlands (35.6%) (*P* < .001). Diabetes mellitus was most common in Indian patients (21.5%) and American patients (10.2%), and less prevalent in Dutch (2.0%) and Austrian (8.0%) patients (*P* < .001). Dyslipidemia was significantly more prevalent in American patients (39.3%) compared to Dutch (4.2%) and Indian (1.6%) patients (*P* < .001) (Table 1). In terms of sex-related disparities, males had a higher rate of connective tissue disease than females (6.3% vs 2.7%; *P* < .001), although it must be noted that these percentages are relative owing to missing values (Table 2).

### Ascending Aortic Dimensions

In the total cohort of ATAAD patients, the mean ascending aortic diameter was 47.5 ± 10.4 mm. Country-specific analysis showed mean diameters of 48.7 ± 11.2 in the United States, 45.2 ± 10.5 in the Netherlands, 48.2 ± 8.1 in Austria, and 53.4 ± 13.8 in India (*P* < .001). When indexed to height, Indian patients had a higher aortic size index compared to their Western counterparts (3.2 ± 0.9 vs 2.8 ± 0.5 for Austria, 2.6 ± 0.6 for the Netherlands, and 2.8 ± 0.7 for the United States; *P* < .001).

Men had a larger mean ascending aortic diameter than women (48.4 ± 10.6 vs 46.0 ± 9.8; *P* < .001). Overall, the ascending aortic diameter was ≤30 mm in 1.8% of patients, 30 to 34 mm in 5.5%, 35 to 39 mm in 12.1%, 40 to 44 mm in 21.5%, 45 to 49 mm in 21.0%, 50 to 54 mm in 18.7%, and ≥55 mm in 19.5%. Notably, the majority of American and Austrian patients presented with diameters of 40 to 54 mm, whereas a higher proportion of Indian patients presented with diameters ≥55 mm (*P* < .001) (Tables 3 and 4 and Figure 2).

**Table 3:** Aortic diameters

Ascending aorta (mm)	Total (N = 1388)	United States (N = 382)	The Netherlands (N = 500)	Austria (N = 435)	India (N = 71)	*P* value	Males (N = 890)	Females (N = 498)	*P* value
Minimum	20.0	27.0	20.0	24.0	30.0	<.001	20.0	24.0	<.001
Maximum	100.0	100.0	90.0	93.3	93.5		100.0	90.0	
Mean ± SD	47.5 ± 10.4	48.7 ± 11.2	45.2 ± 10.5	48.2 ± 8.1	53.4 ± 13.8		48.4 ± 10.6	46.0 ± 9.8	
Median (IQR)	47.0 (41.0-52.0)	48.0 (41.0-53.8)	44.0 (39.0-50.0)	47.0 (43.0-52.0)	51.0 (44.0-60.0)		47.0 (42.0-53.0)	45.0 (40.0-51.0)	

*SD*, Standard deviation; *IQR*, interquartile range.

**Table 4:** Ascending aortic diameter distribution

Aortic diameter	Total (N = 1388)	United States (N = 382)	The Netherlands (N = 500)	Austria (N = 435)	India (N = 71)	*P* value	Male (N = 890)	Female (N = 498)	*P* value
<25 mm	8 (0.6)	0 (0.0)	7 (1.4)	1 (0.2)	0 (0.0)	<.001	5 (0.6)	3 (0.6)	<.001
25-29 mm	17 (1.2)	1 (0.8)	13 (2.6)	1 (0.2)	0 (0.0)		8 (1.5)	9 (1.8)	
30-34 mm	76 (5.5)	24 (6.3)	43 (8.6)	5 (1.1)	4 (5.6)		40 (4.5)	36 (7.2)	
35-39 mm	168 (12.1)	49 (12.8)	78 (15.6)	37 (8.5)	4 (5.6)		93 (10.4)	75 (15.1)	
40-44 mm	298 (21.5)	72 (18.8)	114 (22.8)	99 (22.8)	13 (18.3)		183 (20.6)	115 (23.1)	
45-49 mm	291 (21.0)	61 (16.0)	99 (19.8)	122 (28.0)	9 (12.7)		195 (21.9)	96 (19.3)	
50-54 mm	260 (18.7)	85 (22.3)	66 (13.2)	97 (22.3)	12 (16.9)		173 (19.4)	87 (17.5)	
≥55 mm	270 (19.5)	88 (23.0)	80 (16.0)	73 (16.8)	29 (40.8)		193 (21.7)	77 (15.5)	

Data are presented as n (%).

A multivariate linear regression analysis, corrected for age, sex, height, body surface area, and the presence of hypertension, diabetes, connective tissue disease, and bicuspid aortic valve, identified country of origin as a significant predictors of ascending aortic diameter at dissection. Country of origin also influenced aortic size; patients from Austria (B = 2.42 mm; 95% confidence interval [CI], 1.01-3.85 mm; *P* < .001), the United States (B = 3.11 mm; 95% CI, 1.61-4.62 mm; *P* < .001), and India (B = 7.09 mm; 95% CI, 4.00-10.17 mm; *P* < .001) had larger diameters compared to the reference group (Dutch patients) (Table 5).

**Table 5:** Multivariate linear regression model on predictors for aortic diameter at dissection

Predictor	B (95% CI)	SE	T	*P* value
Constant	43.154 (30.410-55.899)	6.496	6.644	<.001
Age	0.065 (0.019-0.111)	0.023	2.787	.005
Female	−1.839 (−3.175 to −0.502)	0.681	−2.699	.007
Height	−0.018 (−0.102 to 0.066)	0.043	−0.419	.675
BSA	1.885 (−1.583 to 5.353)	1.768	1.066	.286
Country				
The Netherlands				
Austria	2.429 (1.008-3.850)	0.724	3.354	<.001
United States	3.114 (1.609-4.619)	0.767	4.059	<.001
India	7.085 (4.002-10.169)	1.572	4.509	<.001
Diabetes mellitus	−0.473 (−2.695 to 1.749)	1.133	−0.418	.676
Hypertension	−0.061 (−1.279 to 1.157)	0.621	−0.099	.922
Connective tissue disease	0.751 (−2.167 to 3.670)	1.488	0.505	.614
BAV	6.000 (3.461-8.538)	1.294	4.637	<.001

*BSA*, Body surface Area; *BAV*, bicuspid aortic valve.

## Discussion

In this multinational cohort of 1388 surgically treated patients with ATAAD, we found substantial heterogeneity in ascending aortic dimensions at the time of dissection across geographic regions and between sexes. Three principal findings emerge. First, country of origin independently predicts ascending aortic diameter at dissection after adjustment for age, sex, body size, and cardiovascular risk factors. Second, women experience dissection at smaller diameters despite presenting at older age. Third, the majority of dissections occur below currently recommended prophylactic thresholds, reinforcing the limitations of absolute diameter as a universal surrogate for dissection risk.^5^^,^^17^

### Geographic Heterogeneity in Aortic Dimensions at Dissection

The most pronounced geographic differences were observed between South Asian and Western cohorts. Indian patients presented more than a decade earlier than Western patients, dissected at significantly larger ascending aortic diameters, and had significantly higher aortic height indices compared to Western patients. When interpreted alongside available reference data demonstrating smaller baseline ascending aortic dimensions in Indian populations,^9^^,^^18^ these findings suggest that the natural history of thoracic aortic disease may differ across populations. Younger age at presentation combined with larger diameter at dissection raises the possibility of population-specific differences in aortic growth dynamics or wall vulnerability rather than simple extrapolation of Western reference values. Furthermore, the higher aortic height indices in Indian patients compared to Western patients indicate relatively larger aortic growth, possibly underestimating risk if assessed by absolute dimensions alone. This highlights the potential advantage of using indexed measurements, as was also emphasized in recent guidelines.^3^^,^^4^

The mechanisms underlying these geographic disparities remain incompletely understood. The Indian population is known to carry a disproportionately high burden of hypertension and diabetes,^19^ both of which have been associated with adverse aortic remodeling, medial degeneration, and accelerated vascular aging.^20^ Genetic susceptibility, differences in connective tissue composition, and environmental or lifestyle-related exposures also may contribute, although direct evidence remains limited.^21^ Importantly, these observations should be interpreted as hypothesis-generating rather than causal, underscoring the need for dedicated longitudinal and mechanistic studies in diverse populations.

Notably, geographic heterogeneity also was observed within predominantly Caucasian cohorts. Both Austrian and American patients dissected at larger diameters compared to Dutch patients. To our knowledge, such cross-country comparisons within Western populations have not been reported. These findings challenge the implicit assumption that Western cohorts represent a homogeneous reference group for aortic size and risk and suggest that regional phenotype differences may persist even within broadly similar ancestral backgrounds.

### Sex-Specific Differences and Implications for Risk Stratification

Sex-related differences were consistent and clinically relevant. Compared to men, women presented at significantly older ages yet dissected at smaller ascending aortic diameters. This observation aligns with prior studies demonstrating smaller reference aortic dimensions in women, even after indexing to body size or height.^9^^,^^18^^,^^22^ Several investigations have further suggested that women may exhibit higher aortic growth rates than men, potentially contributing to later presentation despite smaller baseline dimensions.^23^

From a clinical perspective, these findings underscore an important limitation of absolute diameter thresholds. Reliance on uniform cut-off values may systematically underestimate the risk of dissection in women and delay consideration of preventive intervention, particularly in those without recognized genetic syndromes. Therefore, our results add to growing evidence that sex-specific reference values and growth trajectories should be integrated into contemporary risk stratification models for thoracic aortic disease.

### Acute Aortic Expansion at the Time of Dissection

A key methodologic consideration is that aortic dimensions measured during acute dissection may overestimate true predissection size. Acute ATAAD alters aortic geometry through wall splitting, intramural hematoma formation, and false-lumen pressurization. Comparative imaging studies in patients with available predissection scans demonstrate that the ascending aorta can acutely increase in diameter at the time of dissection, often on the order of several millimeters, with reported averages commonly in the 5- to 10-mm range, although variability between individuals is substantial.^24^^,^^25^

Importantly, no validated method exists to accurately reconstruct the true predissection diameter or to apply a uniform correction factor at the patient level. Accordingly, we report unadjusted acute-phase measurements, consistent with prior registry-based and surgical series.^5^^,^^25^ Any acute expansion would be expected to bias measured diameters upward rather than downward; therefore, the observation that the majority of patients in our cohort experienced dissection below guideline-recommended thresholds despite this anticipated overestimation further strengthens the conclusion that true predissection diameters are likely even smaller.

### Reframing the “Aortic Size Paradox”

Our findings extend the “aortic size paradox” into a global context. Although individual dissection risk increases steeply with increasing diameter, ascending aortic size follows an approximately bell-shaped population distribution.^17^^,^^26^ Relatively few individuals reach extreme diameters, whereas the largest proportion of the population resides in intermediate ranges. Consequently, most dissections occur numerically at intermediate diameters, not because these diameters are intrinsically low risk but because they contain the largest number of individuals at risk. This distinction between absolute event counts and individual risk is critical for interpreting diameter-stratified event distributions and for understanding why fixed cutoffs can underperform across heterogeneous populations.^17^

This distributional effect also provides a framework for population-specific interpretation. Groups with smaller baseline aortic dimensions may reach a functionally equivalent high-risk zone at lower absolute diameters, effectively entering the upper tail of their population-specific distribution earlier. This concept supports the rationale for considering diameter relative to expected baseline size (eg, indexed measures or *z*-scores) rather than relying exclusively on absolute diameter (population reference frameworks; guideline discussions).

### Clinical Implications

When applying the current class I guideline threshold of ≥55 mm for elective ascending aortic replacement, only 19.5% of patients in our cohort would have met this criterion at the time of presentation. Because aortic diameter may increase during the acute dissection event owing to wall expansion and intramural hematoma formation, the true predissection diameters likely were even smaller. Collectively, these data challenge the universality of the 55-mm paradigm for elective ascending aortic replacement endorsed by contemporary American and European guidelines.^3^^,^^4^ Rather than supporting a single numerical cutoff, our findings favor an individualized approach that incorporates sex- and population-specific reference values and considers the degree of dilation relative to expected baseline size. Although our study does not justify immediate adoption of new numeric thresholds, it provides robust multinational evidence that absolute diameter alone is insufficient to guide preventive strategies on a global scale and may contribute to missed opportunities for timely intervention in vulnerable groups.

### Limitations

Several limitations warrant consideration. First, this study included only surgically treated patients and might not have captured individuals who died before operative intervention, introducing potential selection bias and limiting generalizability to all ATAAD presentations. Second, although patients were categorized by country, within-country ethnic heterogeneity cannot be excluded and may confound interpretation of geographic differences. Third, measurements were derived from acute-phase CT angiography, and no validated method exists to reconstruct true predissection dimensions; however, this bias would be expected to overestimate baseline size. In addition, detailed characterization of the primary intimal tear location was not recorded consistently across all participating cohorts and thus was not included in the present analysis, as the primary aim of this study was the assessment of ascending aortic diameter at the time of dissection. Cross-sectional area indexed to height has been proposed as an alternative measure of aortic size, but it was not evaluated in the present study. This parameter is not routinely used in clinical practice, and current guidelines as well as most risk stratification models continue to rely on absolute aortic diameter. Therefore, maximal aortic diameter was used as the primary measurement in this analysis. Finally, although this represents one of the largest multinational cohorts to date, subgroup sizes, particularly for South Asian patients, remain smaller than Western cohorts, and these findings warrant confirmation in additional datasets.

## Conclusions

In this multinational cohort, ascending aortic dimensions at the time of ATAAD differed significantly by sex and country of origin. ATAAD mostly occurred at diameters below currently recommended prophylactic thresholds, despite measurements obtained during the acute phase. Our findings suggest that a single absolute diameter threshold might not adequately reflect the complexity of dissection risk. The observed variability in dissection diameters across populations suggests that sex- and population-specific reference values merit further investigation.

### Webcast

You can watch a Webcast of this AATS meeting presentation by going to: Xxx.

**Figure:**
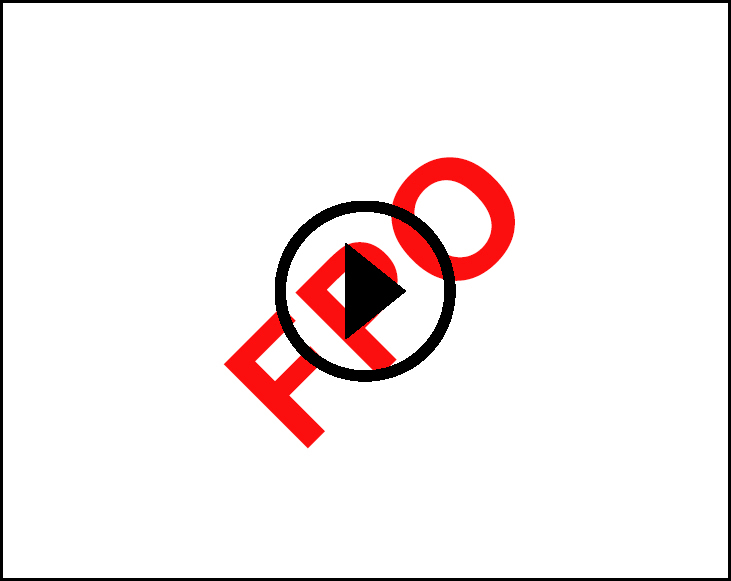

## Conflict of Interest Statement

The authors reported no conflicts of interest.

The *Journal* policy requires editors and reviewers to disclose conflicts of interest and to decline handling or reviewing manuscripts for which they may have a conflict of interest. The editors and reviewers of this article have no conflicts of interest.

## References

[1] Levy D., Sharma S., Farci F., Le J.K. (2025). StatPearls.

[2] Elsayed R.S., Cohen R.G., Fleischman F., Bowdish M.E. (2017). Acute type A aortic dissection. Cardiol Clin.

[3] Czerny M., Grabenwöger M., Berger T. (2024). EACTS/STS guidelines for diagnosing and treating acute and chronic syndromes of the aortic organ. Ann Thorac Surg.

[4] Isselbacher E.M., Preventza O., Hamilton Black J. (2022). 2022 ACC/AHA guideline for the diagnosis and management of aortic disease: a report of the American heart Association/American College of Cardiology Joint Committee on Clinical Practice Guidelines. Circulation.

[5] Pape L.A., Tsai T.T., Isselbacher E.M. (2007). Aortic diameter >or = 5.5 cm is not a good predictor of type A aortic dissection: observations from the International Registry of Acute aortic Dissection (IRAD). Circulation.

[6] Ansari A., Syed K., Baig M. (2024). Ethnic differences in ascending aorta dimensions and dilatation rates: a systematic review. Cureus.

[7] Vandroux D., Aboyans V., Houehanou Y.C. (2022). Normal values of proximal aorta diameters in healthy Sub Saharan Africans: the TAHES study. Echocardiography.

[8] Hu X., Lin Z., Li Y. (2023). Comparisons of two-dimensional echocardiographic aortic dimensions between Chinese, Japanese, and Europeans. J Thorac Imaging.

[9] Bacour N., Idhrees M., Samraj J. (2026). Ethnic variation in thoracic aortic dimensions in the general population: a comparison between Indian and Dutch populations. Open Heart.

[10] Patel M., Abatcha S., Uthman O. (2022). Ethnic differences between South Asians and White Caucasians in cardiovascular disease-related mortality in developed countries: a systematic literature review. Syst Rev.

[11] Wu J., Zeng W., Li X. (2023). Aortic size distribution among normal, hypertension, bicuspid, and Marfan populations. Eur Heart J Imaging Methods Pract.

[12] Rylski B., Desjardins B., Moser W., Bavaria J.E., Milewski R.K. (2014). Gender-related changes in aortic geometry throughout life. Eur J Cardiothorac Surg.

[13] Jasper A., Harshe G., Keshava S., Kulkarni G., Stephen E., Agarwal S. (2014). Evaluation of normal abdominal aortic diameters in the Indian population using computed tomography. J Postgrad Med.

[14] Venkatesan S., Kumar G.P., Nenwani D., Swaminathan N., Shankar G.R., Paul G.J. (2020). Measurement of aortic root dimensions by transthoracic echocardiogram in normal Indian population. J Indian Acad Echocardiogr Cardiovasc Imaging.

[15] Elefteriades J.A., Mukherjee S.K., Mojibian H. (2020). Discrepancies in measurement of the thoracic aorta: JACC review topic of the week. J Am Coll Cardiol.

[16] Zafar M.A., Li Y., Rizzo J.A. (2018). Height alone, rather than body surface area, suffices for risk estimation in ascending aortic aneurysm. J Thorac Cardiovasc Surg.

[17] Paruchuri V., Salhab K.F., Kuzmik G. (2015). Aortic size distribution in the general population: explaining the size paradox in aortic dissection. Cardiology.

[18] Idhrees M., Bacour N., Dolmaci O., Samraj J., Velayudhan B., Grewal N. (2026). Establishing reference values for aortic dimensions in the general Indian population and implications for global standards. Indian J Thorac Cardiovasc Surg.

[19] Kishore J., Tripathi N., Debnath A., Gupta S., Babu B.V. (2025). Prevalence of diabetes, hypertension, and their risk factors among the migrant tribal community of Delhi: a cross-sectional study. Int J Noncommun Dis.

[20] Herzog M.J., Müller P., Lechner K. (2025). Arterial stiffness and vascular aging: mechanisms, prevention, and therapy. Signal Transduct Target Ther.

[21] Bacour N., Theijse R.T., Grewal N., Klautz R. (2025). Racial and Ethnic Disparities in the Presentation and Outcome of Patients with Thoracic Aortic Aneurysms. J Cardiovasc Dev Dis.

[22] Carrero M.C., Matta M.G., Constantin I., Masson G., Asch F.M. (2025). Sex-specific considerations in defining aortic dilation: findings from the MATEAR study. Arch Cardiol Mex.

[23] Cheung K., Boodhwani M., Chan K.L., Beauchesne L., Dick A., Coutinho T. (2017). Thoracic aortic aneurysm growth: role of sex and aneurysm etiology. J Am Heart Assoc.

[24] Rylski B., Blanke P., Beyersdorf F. (2014). How does the ascending aorta geometry change when it dissects?. J Am Coll Cardiol.

[25] Ziganshin B.A., Zafar M.A., Elefteriades J.A. (2019). Descending threshold for ascending aortic aneurysmectomy: is it time for a "left-shift" in guidelines?. J Thorac Cardiovasc Surg.

[26] Davies R.R., Goldstein L.J., Coady M.A. (2002). Yearly rupture or dissection rates for thoracic aortic aneurysms: simple prediction based on size. Ann Thorac Surg.

